# Supplementary material for: A fault-intrusion-tolerant system and deadline-aware algorithm for scheduling scientific workflow in the cloud
Source: PeerJ Comput Sci. 2021 Nov 2;7:e747. doi: 10.7717/peerj-cs.747 (PMC8576554; doi:10.7717/peerj-cs.747)
Supplement: Supplemental Information 6 [file peerj-cs-07-747-s006.zip › Myproject1.1/docs/org/workflowsim/WorkflowPlanner.html]

WorkflowPlanner


JavaScript is disabled on your browser.


- Overview
- Package
- Class
- Use
- Tree
- Deprecated
- Index
- Help

- Prev Class
- Next Class

- Frames
- No Frames

- All Classes

- Summary:
- Nested |
- Field |
- Constr |
- Method

- Detail:
- Field |
- Constr |
- Method


org.workflowsim

## Class WorkflowPlanner

- java.lang.Object
- - org.cloudbus.cloudsim.core.SimEntity
  - - org.workflowsim.WorkflowPlanner

- All Implemented Interfaces:
  :   java.lang.Cloneable

  ---

    

  ```
  public class WorkflowPlanner
  extends SimEntity
  ```

  WorkflowPlanner supports dynamic planning. In the future we will have global
  and static algorithm here. The WorkflowSim starts from WorkflowPlanner.

  Since:
  :   WorkflowSim Toolkit 1.0

- - ### Field Summary

    Fields

    | Modifier and Type | Field and Description |
    | --- | --- |
    | `protected WorkflowParser` | `parser` The workflow parser. |
    | `protected java.util.List<Task>` | `taskList` The task list. |

    - ### Fields inherited from class org.cloudbus.cloudsim.core.SimEntity

      `FINISHED, HOLDING, RUNNABLE, WAITING`
  - ### Constructor Summary

    Constructors

    Constructor and Description

    `WorkflowPlanner(java.lang.String name)`

    Created a new WorkflowPlanner object.

    `WorkflowPlanner(java.lang.String name,
    int schedulers)`
  - ### Method Summary

    Methods

    | Modifier and Type | Method and Description |
    | --- | --- |
    | `protected void` | `finishExecution()` Send an internal event communicating the end of the simulation. |
    | `ClusteringEngine` | `getClusteringEngine()` Gets the clustering engine |
    | `int` | `getClusteringEngineId()` Gets the clustering engine id |
    | `java.util.List<Task>` | `getTaskList()` Gets the task list. |
    | `WorkflowEngine` | `getWorkflowEngine()` Gets the workflow engine |
    | `int` | `getWorkflowEngineId()` Gets the workflow engine id |
    | `WorkflowParser` | `getWorkflowParser()` Gets the workflow parser |
    | `void` | `processEvent(SimEvent ev)` Processes events available for this Broker. |
    | `protected void` | `processOtherEvent(SimEvent ev)` Overrides this method when making a new and different type of Broker. |
    | `protected void` | `setTaskList(java.util.List<Task> taskList)` Sets the task list. |
    | `void` | `shutdownEntity()` This method is invoked by the `Simulation` before the simulation finishes. |
    | `void` | `startEntity()` This method is invoked by the `Simulation` class when the simulation is started. |

    - ### Methods inherited from class org.cloudbus.cloudsim.core.SimEntity

      `cancelEvent, clone, getEventBuffer, getId, getName, getNextEvent, getNextEvent, getState, numEventsWaiting, numEventsWaiting, pause, run, schedule, schedule, schedule, schedule, scheduleFirst, scheduleFirst, scheduleFirst, scheduleFirst, scheduleFirstNow, scheduleFirstNow, scheduleFirstNow, scheduleFirstNow, scheduleNow, scheduleNow, scheduleNow, scheduleNow, selectEvent, send, send, send, send, sendNow, sendNow, sendNow, sendNow, setEventBuffer, setId, setState, waitForEvent`
    - ### Methods inherited from class java.lang.Object

      `equals, finalize, getClass, hashCode, notify, notifyAll, toString, wait, wait, wait`

- - ### Field Detail


    - #### taskList

      ```
      protected java.util.List<Task> taskList
      ```

      The task list.


    - #### parser

      ```
      protected WorkflowParser parser
      ```

      The workflow parser.
  - ### Constructor Detail


    - #### WorkflowPlanner

      ```
      public WorkflowPlanner(java.lang.String name)
                      throws java.lang.Exception
      ```

      Created a new WorkflowPlanner object.

      Parameters:
      :   `name` - name to be associated with this entity (as required by
          Sim\_entity class from simjava package)

      Throws:
      :   `java.lang.Exception` - the exception


    - #### WorkflowPlanner

      ```
      public WorkflowPlanner(java.lang.String name,
                     int schedulers)
                      throws java.lang.Exception
      ```

      Throws:
      :   `java.lang.Exception`
  - ### Method Detail


    - #### getClusteringEngineId

      ```
      public int getClusteringEngineId()
      ```

      Gets the clustering engine id

      Returns:
      :   clustering engine id


    - #### getClusteringEngine

      ```
      public ClusteringEngine getClusteringEngine()
      ```

      Gets the clustering engine

      Returns:
      :   the clustering engine


    - #### getWorkflowParser

      ```
      public WorkflowParser getWorkflowParser()
      ```

      Gets the workflow parser

      Returns:
      :   the workflow parser


    - #### getWorkflowEngineId

      ```
      public int getWorkflowEngineId()
      ```

      Gets the workflow engine id

      Returns:
      :   the workflow engine id


    - #### getWorkflowEngine

      ```
      public WorkflowEngine getWorkflowEngine()
      ```

      Gets the workflow engine

      Returns:
      :   the workflow engine


    - #### processEvent

      ```
      public void processEvent(SimEvent ev)
      ```

      Processes events available for this Broker.

      **Specified by:**
      :   `processEvent` in class `SimEntity`

      Parameters:
      :   `ev` - a SimEvent object


    - #### processOtherEvent

      ```
      protected void processOtherEvent(SimEvent ev)
      ```

      Overrides this method when making a new and different type of Broker.
      This method is called by `#body()` for incoming unknown tags.

      Parameters:
      :   `ev` - a SimEvent object


    - #### finishExecution

      ```
      protected void finishExecution()
      ```

      Send an internal event communicating the end of the simulation.


    - #### shutdownEntity

      ```
      public void shutdownEntity()
      ```

      **Description copied from class: `SimEntity`**

      This method is invoked by the `Simulation` before the simulation finishes. If you want
      to save data in log files this is the method in which the corresponding code would be placed.

      **Specified by:**
      :   `shutdownEntity` in class `SimEntity`


    - #### startEntity

      ```
      public void startEntity()
      ```

      **Description copied from class: `SimEntity`**

      This method is invoked by the `Simulation` class when the simulation is started. This
      method should be responsible for starting the entity up.

      **Specified by:**
      :   `startEntity` in class `SimEntity`


    - #### getTaskList

      ```
      public java.util.List<Task> getTaskList()
      ```

      Gets the task list.

      Type Parameters:
      :   `T` - the generic type

      Returns:
      :   the task list


    - #### setTaskList

      ```
      protected void setTaskList(java.util.List<Task> taskList)
      ```

      Sets the task list.

      Type Parameters:
      :   `T` - the generic type

      Parameters:
      :   `cloudletList` - the new task list


- Overview
- Package
- Class
- Use
- Tree
- Deprecated
- Index
- Help

- Prev Class
- Next Class

- Frames
- No Frames

- All Classes

- Summary:
- Nested |
- Field |
- Constr |
- Method

- Detail:
- Field |
- Constr |
- Method
